# Soil Microbial Resource Limitations and Community Assembly Along a *Camellia oleifera* Plantation Chronosequence

**DOI:** 10.3389/fmicb.2021.736165

**Published:** 2021-12-02

**Authors:** Hang Qiao, Longsheng Chen, Yajun Hu, Chenghua Deng, Qi Sun, Shaohong Deng, Xiangbi Chen, Li Mei, Jinshui Wu, Yirong Su

**Affiliations:** ^1^Key Laboratory of Agro-ecological Processes in Subtropical Region, Institute of Subtropical Agriculture, Chinese Academy of Sciences, Changsha, China; ^2^College of Resource and Environment, University of Chinese Academy of Sciences, Beijing, China; ^3^Research Institute of Economic Forest and Fruit (Research Institute of Oil Tea Camellia), Hunan Academy of Forestry, Changsha, China; ^4^College of Agronomy, Hunan Agricultural University, Changsha, China; ^5^College of Horticulture and Forestry Sciences/Hubei Engineering Technology Research Center for Forestry Information, Huazhong Agricultural University, Wuhan, China

**Keywords:** soil microbial limitation, community assembly, stand age, planted forest, *Camellia oleifera*

## Abstract

Understanding soil microbial element limitation and its relation with the microbial community can help in elucidating the soil fertility status and improving nutrient management of planted forest ecosystems. The stand age of a planted forest determines the aboveground forest biomass and structure and underground microbial function and diversity. In this study, we investigated 30 plantations of *Camellia oleifera* distributed across the subtropical region of China that we classified into four stand ages (planted <9 years, 9–20 years, 21–60 years, and >60 years age). Enzymatic stoichiometry analysis showed that microbial metabolism in the forests was mainly limited by C and P. P limitation significantly decreased and C limitation slightly increased along the stand age gradient. The alpha diversity of the soil microbiota remained steady along stand age, while microbial communities gradually converged from scattered to clustered, which was accompanied by a decrease in network complexity. The soil bacterial community assembly shifted from stochastic to deterministic processes, which probably contributed to a decrease in soil pH along stand age. Our findings emphasize that the stand age regulated the soil microbial metabolism limitation and community assembly, which provides new insight into the improvement of C and P management in subtropical planted forest.

## Introduction

Planted forests, established by planting and/or deliberate seeding, provide critical ecosystem services, such as carbon storage, soil conservation, and wood production (FAO, 2015). Planted forests have been estimated to have increased globally from 277.9 million ha to 320 million ha between 2015 and 2020 (Nepal et al., 2019). Compared to natural forests, planted forests, which are generally established with the aim to restore plant cover on agricultural and mined lands, are characterized by a lower plant diversity (Martínez-Jauregui et al., 2016). According to the widely accepted resource diversity hypothesis, plant communities with a high diversity support higher soil microbial activity and diversity owing to a diverse and complex organic substrate input from the various species planted. For example, the soil microbial activity in a mixed hornbeam and ironwood forest was found to be higher than in an ash planted forest (Kooch et al., 2018). The microbial diversity in a natural hygrophilic deciduous mixed forest was higher than that in the poplar planted forest (Vitali et al., 2016). The investigation of soil microbial processes in planted forests is expected to deepen our understanding of why the microbes show a low activity and diversity in such forests.

Soil microbial metabolic limitation, which reflects the nutrient demands of soil microorganisms for microbial metabolic processes, can be analyzed based on microbial enzyme activity as indicated by eco-enzymatic stoichiometry (Sinsabaugh et al., 2015). Previous studies have reported that soil microbial carbon (C) limitation is common in terrestrial ecosystems (Schimel and Weintraub, 2003), whereas microbial nitrogen (N) and phosphorus (P) limitations are more generally found in grassland and wetland ecosystems (Hill et al., 2018; Yang et al., 2020). Soil microbial metabolic limitation is influenced by multiple environmental factors, including climatic and plant-related factors. Microbial C limitation reportedly decreases with increasing precipitation in the Loess Plateau region (Cui et al., 2019). Soil microbial P limitation increases during forest succession from coniferous to broad-leaf forest due to substantial competition for P in the later stages of forest succession (Huang et al., 2013). Thus, the identification of the variation in soil microbial metabolic limitation in a specific forest ecosystem will improve our knowledge of the soil biogeochemical constraints in the system.

Stand age is a primary driver of forest structure and function, including plant net primary productivity, carbon storage (Pregitzer and Euskirchen, 2004), and the soil microbial community (Kang et al., 2018). A previous study showed that soil bacterial alpha diversity increased linearly with increasing stand age in *Caragana liouana* plantations (Na et al., 2018), whereas it exhibited a nonlinear pattern along stand age in *Hevea brasiliensis* plantations (Zhou et al., 2017). The inconsistency of microbial diversity patterns is usually attributed to the plant species involved and the soil physicochemical properties. For example, *Alnus cremastogyne*, as a pioneer species, gradually decreases the soil pH through the secretion of organic acids during tree growth, thus increasing soil bacterial diversity (Sun et al., 2018). The progressive accumulation of lignin-rich litter from oak trees on the soil surface along stand age provides a moist soil microclimate that supports the growth of anaerobic bacterial species (Canessa et al., 2020). Additionally, forest stand age also affects microbial metabolic limitation *via* influencing soil nutrients. A study in a Douglas-fir forest revealed that microbial N limitation increased with stand age (Vittori Antisari et al., 2018). In Eucalyptus plantations, microbial metabolism was mainly limited by P and increased with stand age (Fan et al., 2015). Thus, the effect of stand age on the soil microbial diversity depends on the forest species.

Shifts in a microbial community along ecological successions are controlled by microbial assembly processes (Stegen et al., 2012). Deterministic processes result from environmental abiotic and biotic filtering that shapes species abundances, whereas stochastic processes reflect changes in species abundances by random processes, such as ecological drift and dispersal (Dini-Andreote et al., 2015). The relative importance of these two processes is determined by various factors, including spatial variation (Gao et al., 2019), ecosystem succession (Måren et al., 2018), and environmental disturbances (Ferrenberg et al., 2013). For example, the soil bacterial community assembly generally shifts with the spatial scale, with stochastic processes dominating at small spatial scales and deterministic processes dominating at larger scales (Feng et al., 2019). In a subtropical forest succession, deterministic processes have been found to govern the soil fungal assembly in the early succession stage, whereas both deterministic and stochastic processes were predominant in later succession stages (Chai et al., 2016). However, the factors influencing soil microbial assembly on a short time scale, such as stand age, remain largely unknown.

*Camellia oleifera*, one of the four major woody oil plants in the world, is widely planted in the subtropical zone of South China (Jin and Ning, 2012). The planting area of *C. oleifera* increases at the rate of 0.1 million ha annually and covers about 4.5 million ha as of 2019 (State Forestry Bureau, 2009). Besides the economic purpose of oil production, *C. oleifera* is planted as a pioneer species to colonize acid, infertile soils for ecological restoration (Yang et al., 2016). However, continuous product harvesting has been shown to decrease soil fertility in *C. oleifera* forests. Under infertile conditions, soil microbes compete with plants for nutrients, such as N and P, resulting in low tea quality and oil yield (Liu et al., 2017). Understanding soil microbial metabolism and community dynamics is expected to provide useful information for soil fertility management. In this study, field investigation experiments were conducted to examine the effects of stand age on soil microbial metabolic limitation and community assembly in *C. oleifera* plantations. We hypothesized that (i) microbial C limitation would increase with stand age due to a decrease in soil fertility along stand age, and (ii) deterministic processes rather than random processes determine the soil bacterial community assembly along stand age because *C. oleifera* plants secrete organic acids from their roots and thus decrease soil pH.

## Materials and Methods

### Field Experimental Design and Sample Collection

**Figure 1:**
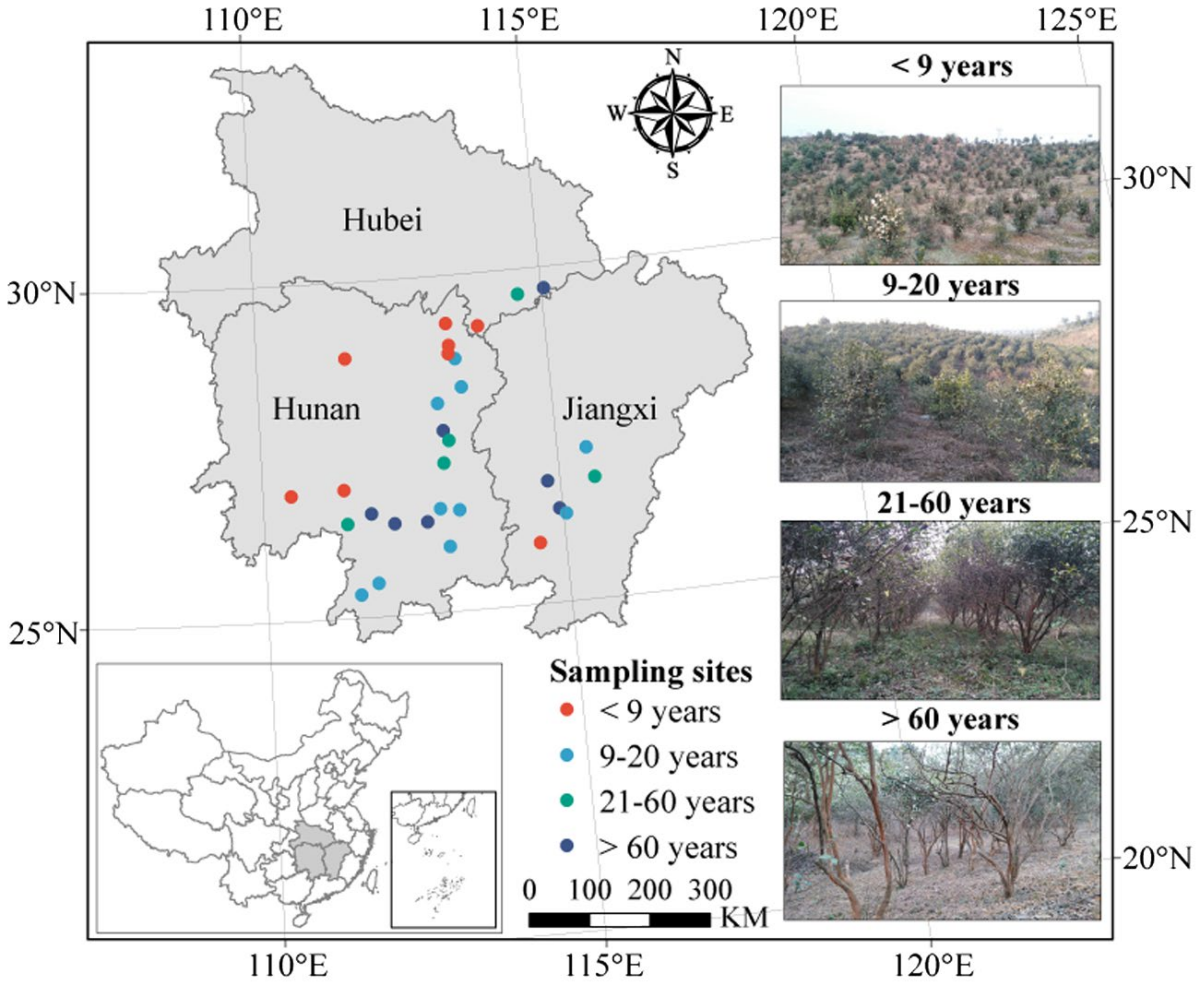
Geographical locations of the sampling sites.

We collected soil samples in plantations of four stand ages, classified as young (planted <9 years ago), near-mature (planted 9–20 years ago), mature (planted 21–60 years ago), or over-mature (planted >60 years ago), based on the space-for-time substitution method, during from December 2017 and January 2018. In total, 30 *C. oleifera* plantations across South China [from a latitude of 25°21′ to 29°42′ (N) and a longitude of 110°28′ to 115°34′ (E)] were investigated (Figure 1). Three independent 100 m^2^ (10 m × 10 m) plots within each plantation were randomly designated for soil sampling. Twelve soil cores taken to a depth of 15 cm were randomly collected within the drip line of a tree in each plot as composite soil samples. The samples were transported to the laboratory in ice boxes. After homogenization, the soil samples were passed through a 2 mm sieve to remove plant residues and stones and were then divided into three subsamples. One subsample was stored at −80°C for microbial DNA analysis, one was air-dried for physicochemical analysis, and one was stored at 4°C until determination of the soil microbial biomass and enzyme activities.

### Determination of Soil Enzyme Activities and Quantification of Microbial Metabolic Limitation

The activities of five extracellular enzymes, including two C acquisition enzymes [β-1,4-glucosidase (BG) and β-D-cellobiosidase (CBH)], two N acquisition enzymes [L-leucine aminopeptidase (LAP) and β-N-acetylglucosaminidase (NAG)], and P acquisition enzyme [acid phosphatase (AP)], were determined using published standard fluorometric techniques (Supplementary Table S1; Saiya-Cork et al., 2002). Briefly, 1 g of fresh soil was homogenously suspended in 125 ml of 50 mm sodium acetate trihydrate (CAS). Then, 250 μl of CAS, 200 μl of the soil suspensions, 50 μl of 10 M methylumbelliferyl solution, and 50 μl of substrate were added into each well of 96-well microplates and incubated at 25°C in the dark for 4 h. Then, 10 μl of 1 M NaOH was added to stop the reaction. Fluorescence was determined using a Synergy H4 multimode microplate reader (Infinite 200 Pro, Tecan, Switzerland).

Soil microbial metabolic limitation was evaluated by two different methods. The first method uses a scatter plot of soil enzymatic activities, represented by the N:P versus C:N enzymatic activity ratios (Sinsabaugh et al., 2009). This quadrantal diagram provides information regarding four types of microbial metabolic limitation, that is, P limitation, N limitation, C&P limitation, and N&P limitation (Schmidt et al., 2016). The second method uses vector analysis of the enzymatic stoichiometry and considers the lowest kurtosis for vector angle, as suggested by Moorhead et al. (2016). Vector length (L) was used to describe the microbial C limitation (i.e., increased vector L suggests increased microbial C limitation), whereas the vector angle (A°) was used to indicate microbial N and P limitation, with vector A > 45° and < 45° representing P limitation and N limitation, respectively. Vector L and vector A were calculated using the following formulae (Moorhead et al., 2016):


VectorL=lnBG+CBH/lnNAG+LAP^2+lnBG+CBH/lnAP^2

$$Vector\;A=\mathrm{Degrees}\left(\mathrm{ATAN}2\left(\begin{array}{l} \left(\ln\left[\mathrm{BG}+\mathrm{CBH}\right]/\mathrm{lnAP}\right),\\ \left(\ln\left[\mathrm{BG}+\mathrm{CBH}\right]/\ln\left[\mathrm{NAG}+\mathrm{LAP}\right]\right) \end{array}\right)\right)$$

### DNA Extraction, PCR, and High-Throughput Sequencing

Soil DNA was extracted using the PowerSoil DNA Isolation Kit (MoBIo Laboratories, Carlsbad, CA, United States), according to the manufacturer’s manual. DNA quality was evaluated using a NanoDrop ND-2000 spectrophotometer (Thermo Scientific, United States). The 16S rRNA gene V3-V4 region was amplified using the primer set 343F (5′-TACGGRAGGCAGCAG-3′)/798R (5′-AGGGTATCTAATCCT-3′; Nossa et al., 2010). A unique 8-mer tag was designed and linked to the 5′ end of each primer to allow sample identification in multiplex samples. PCRs were run using 25 μl reaction mixtures containing 12.5 μl of 2× KAPA HiFi HotStart ReadyMix, 5 μl of each primer (1 μm), and 2.5 μl of diluted DNA (5 ng/μl) and the following thermal cycling program: 95°C for 3 min, 30 cycles of 95°C for 30 s, 60°C for 30 s, and 72°C for 30 s, and 72°C for 10 min. PCR products were purified using AMPure XP Beads (Beckman Coulter, United Kingdom). Purified PCR amplicons were combined at equimolar concentrations after quantification using the Qubit dsDNA HS Assay Kit (Invitrogen, United States) for sequencing library construction. The library was sequenced on an Illumina MiSeq platform (2 × 300 bp) at Shanghai Hanyu Biotech (Shanghai, China).

Pair-end raw reads were assembled, screened, and trimmed using the mothur software (v.1.36.1; Schloss et al., 2009). Briefly, stringent quality-based trimming was first used to minimize sequencing error effects (Kunin et al., 2010). Sequences were removed based on the following criteria: average quality score of 50-bp windows <25, homopolymers of more than eight bases, primer sequence, ambiguous base call, and read length < 200 bp. The remaining sequences were sorted by tag sequence and then checked for chimeras using the “screen.seqs” command. The USEARCH algorithm with a 97% identity threshold was adopted for operational taxonomic units (OTUs) clustering (Edgar, 2010). Representative sequences of OTUs were used for BLAST searches against the Greengenes Database (release 13.5) for taxonomic annotation (DeSantis et al., 2006). Each soil bacterial 16S gene sequence was rarified to the same sequencing depth (6,996 sequences per sample) for community analysis. The raw reads were deposited in the NCBI Sequence Read Archive under accession number PRJNA577346.

### Soil Bacterial Community Assembly Processes and Co-occurrence Network Analysis

Soil bacterial community assembly was inferred based on deterministic and stochastic processes. The β-nearest taxon index (βNTI) was calculated to discriminate these processes using the “picante” package in R. Soil bacterial community assembly was inferred based on deterministic and stochastic processes. The βNTI was calculated to discriminate these processes using the “picante” package in R. Values of |βNTI| > 2 indicated that deterministic processes are dominant, and βNTI > +2 and βNTI < −2 reflected variable selection and homogeneous selection, respectively. However, |βNTI| values < 2 indicated stochastic processes are dominant, the Raup-Crick metric (RCbray) based on Bray-Curtis distance were calculated to distinguish these stochastic scenarios, including homogenizing dispersal, dispersal limitation, and undominated. The relative influence of homogenizing dispersal and dispersal limitation denoted by |βNTI| < 2 but RCbray < −0.95 and |βNTI| < 2 but RCbray > +0.95, respectively. The scenario of |βNTI| < 2 but |RCbray| < 0.95 indicated the undominated fraction (Stegen et al., 2015). Relationships between βNTI values and soil properties based on Euclidean distance matrices were evaluated using a Mantel test with 999 permutations using the “vegan” package in R.

Soil bacterial co-occurrence networks were constructed based on the sparse correlations for compositional data (sparCC) correction, using bacterial OTU profiles. The random matrix theory-based method was first used to assess the threshold value for the correlation coefficients between the OTUs, using the “RMThreshold” package in R. Correlation coefficients with an absolute value of ≥0.5924 and *p* < 0.05 were considered for co-occurrence networks analysis (Supplementary Figure S1). Then, we constructed a global co-occurrence network using all selected significant species-species (OTU-OTU) associations using the “igraph” package (Csardi and Nepusz, 2006). Sub-networks were extracted from the global network to identify topological network features for each soil sample using the “induced_subgraph” function. Topological features, including transitivity, average degree, betweenness centrality, average path length, and density, were calculated. Additionally, the topological characteristic of each node in the network was assessed based on within-module connectivity (Zi) and among-module connectivity (Pi). All species were divided into four groups, that is, module hubs (Zi > 2.5), network hubs (Zi > 2.5 and Pi > 0.62), peripherals (Zi < 2.5 and Pi < 0.62), and connectors (Pi > 0.62; Olesen et al., 2007). The species identified as module hubs, network hubs, or connectors were suggested as keystone species. Network visualization and modular analysis were achieved using the interactive Gephi 0.9.2 platform (Bastian et al., 2009).

### Measurements of Soil Physicochemical Properties

Soil pH was measured in a 1:2.5 (v:v) soil:water suspension with a digital pH meter (Mettler-Toledo 320, China; Bao, 2000). Soil organic carbon (SOC) was determined using the Walkley-Black method (Walkley and Black, 1934). Soil total N (TN) was measured by flow injection analysis based on the Kjeldahl method (Bremner, 1960). Soil total P (TP) and Olsen-P were determined using the ammonium molybdate method on an UV spectrophotometer (UV-2550, Shimadzu, Japan) at 700 nm (Olsen and Sommers, 1982). TP was extracted by NaOH digestion, and Olsen-P was extracted using 0.5 M NaHCO_3_.

### Statistical Analysis

Significant differences in soil physicochemical properties, vector characteristics, enzyme activities, enzymatic stoichiometry, bacterial diversity indices, and βNTI values across stand ages were determined by ANOVA using the “aov” function. Linear regressions were used to examine relationships between vector L, vector A, and soil physicochemical properties. A simple clustering heatmap of dominant soil bacterial species was produced using the “heatmap” package. Principal co-ordinates analysis (PCoA) was carried out to detect bacterial community dissimilarity based on the Bray-Curtis distance. The multivariate dispersion index analysis (MVDISP) and permutational analysis of multivariate dispersions (PERMDISP) were adopted to examine the significant differences in bacterial communities among stand ages using the “vegan” package in R. Redundancy analysis (RDA) was performed to investigate the effect of soil physicochemical properties on soil bacterial community structure using the “vegan” package. Data visualization was achieved using the “ggplot2” package. Phylogenetic tree was annotated and visualized in iTOL website^1^ (Letunic and Bork, 2019). Statistical analysis was conducted in R 3.6.1.^2^

## Results

### Soil Enzymatic Activities and Soil Microbial Metabolic Limitations Along a Stand Age Gradient

**Figure 2:**
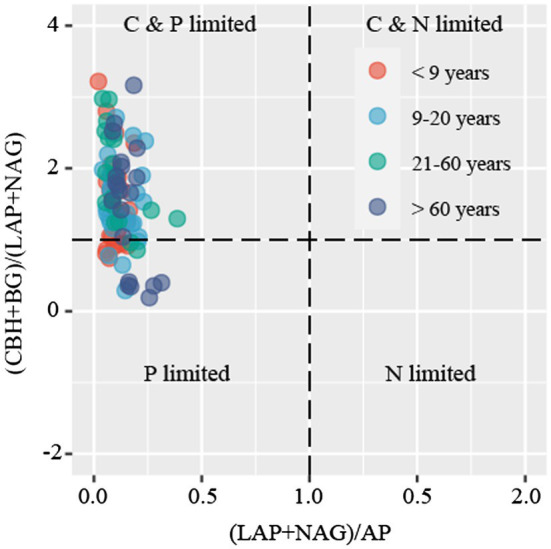
Scatter plots of soil enzymatic stoichiometry for the studied sites. Dots with different colors represent different stand ages.

**Figure 3:**
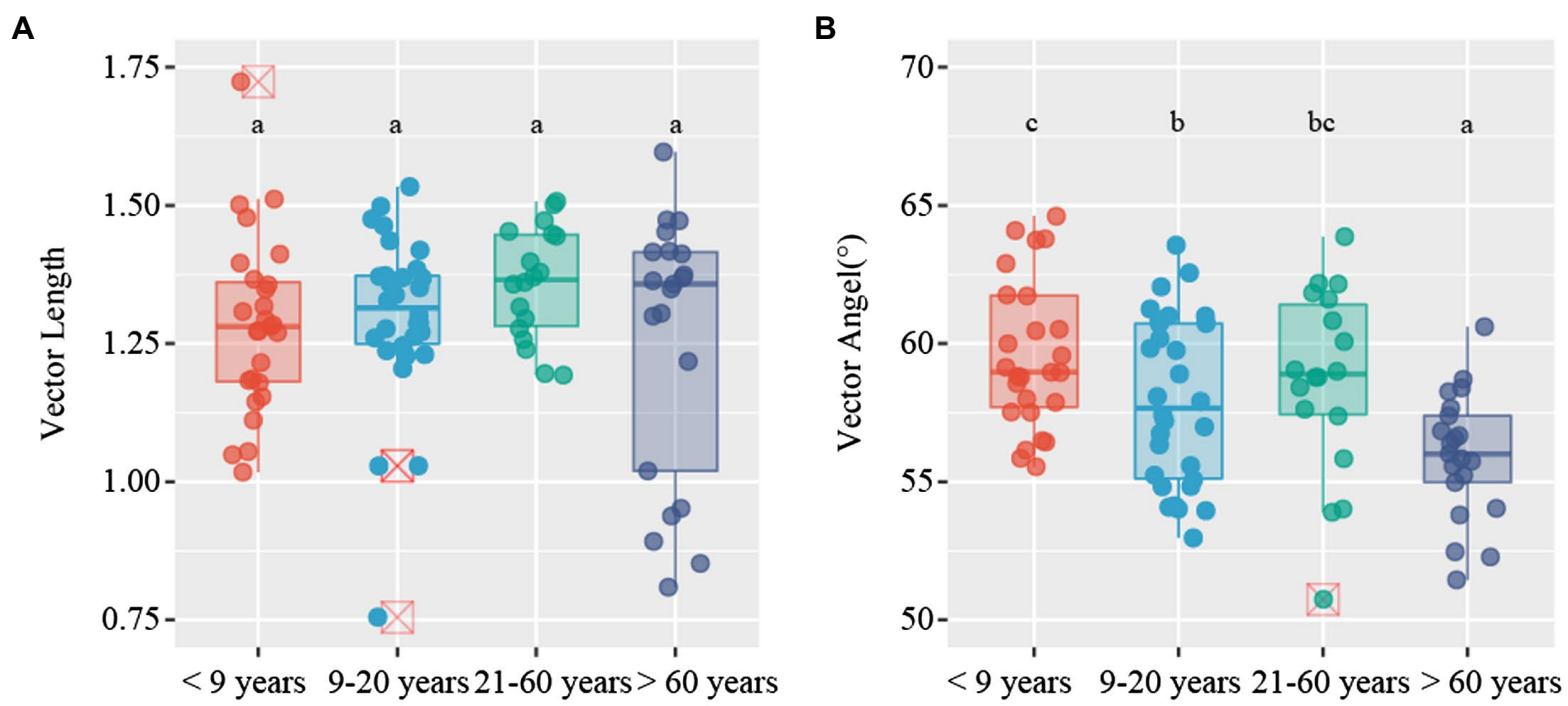
Variations in vector characteristics for *Camellia oleifera* plantations of different stand ages. **(A)** vector length; **(B)** vector angle. Different letters denote significant differences among stand ages (*p* < 0.05).

C-acquiring (CBH and BG), N-acquiring (LAP and NAG), and P-acquiring (AP) enzyme activities tended to increase along stand age (Supplementary Figure S2). The enzymatic stoichiometry of N:P, represented by (LAP + NAG):AP, showed a gradually increasing trend along stand age, whereas stand age had no effect on the enzymatic ratios of C:N and C:P, indicated by (BG + CBH):(LAP + NAG) and (BG + CBH):AP, respectively (Supplementary Figure S3). The scatter plot of enzymatic stoichiometry showed that all soil samples tested were P-limited or co-C- and P-limited (Figure 2). Vector L, as an indicator of microbial C limitation, tended to slightly increase along stand age (Figure 3A). In contrast, vector A, indicating microbial N and P limitations, significantly decreased with increasing stand age (Figure 3B). Soil parameters, including SOC, TN, and TP, were negatively related to vector A, whereas TN was positively related to vector L (Supplementary Figure S4).

### Soil Bacterial Diversity and Community Structure

**Figure 4:**
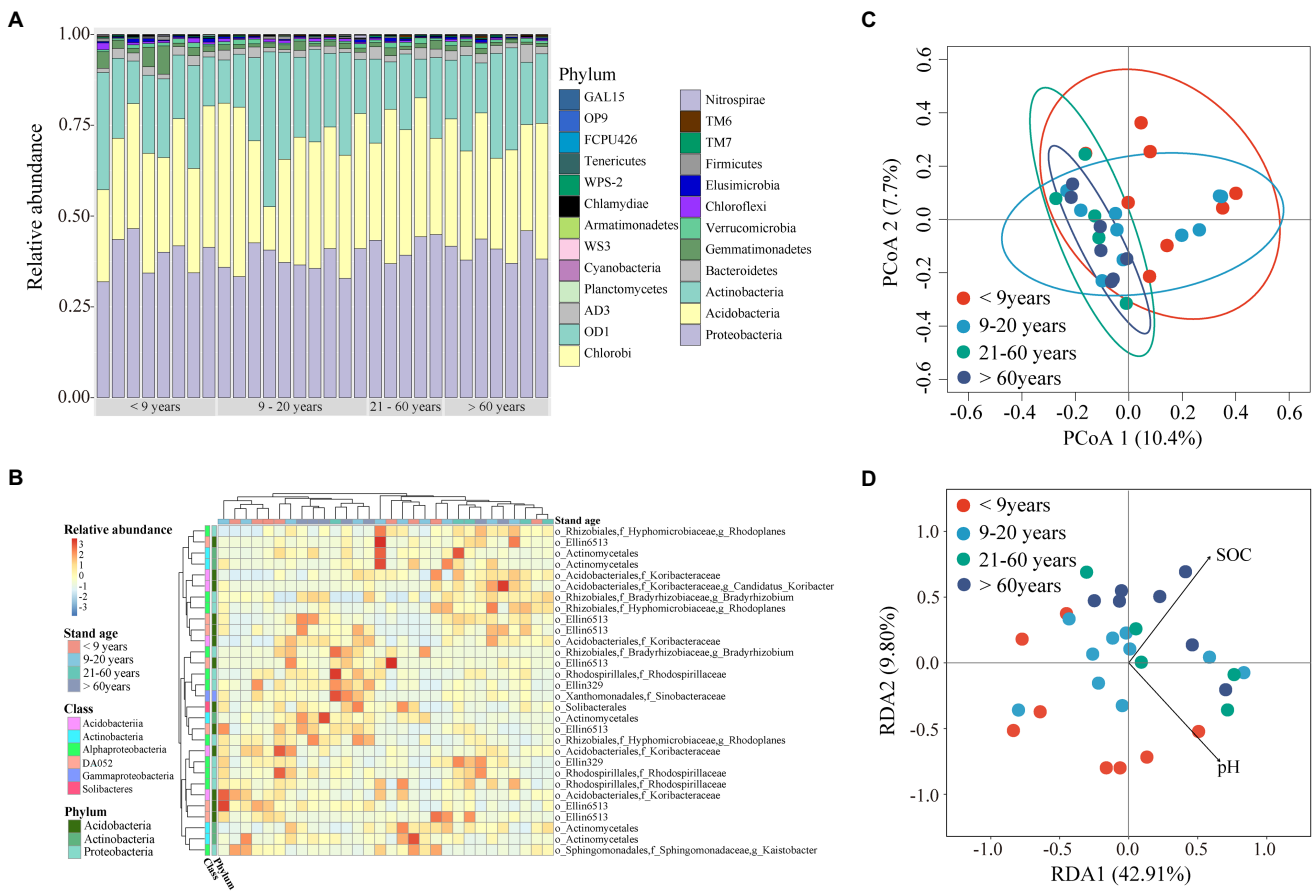
Bacterial community composition along stand age. **(A)** Relative bacterial abundances at the phylum level. **(B)** Heatmap of relative abundances of the 30 most abundant operational taxonomic units (OTUs). **(C)** Principal co-ordinates analysis (PCoA) for bacterial community structure based on Bray-Curtis dissimilarity. **(D)** Redundancy analysis (RDA) showing the effects of different factors on bacterial communities. SOC, soil organic carbon; TN, total nitrogen; TP, total phosphorous; Olsen-P, Olsen phosphorous.

Soil bacterial alpha diversity, including richness and the Shannon index, showed a decreasing trend in near-mature plantations (planted 9–20 years ago) when compared to plantations of the other three stand ages (Supplementary Figure S5). To better characterize bacterial community, we established phylogenetic tree using the top 100 OTUs with high relative abundance. The results showed these dominant OTUs were mainly affiliated within phyla Proteobacteria, Acidobacteria, and Actinobacteria, with a relative abundance of 38–42%, 31–34% and 18–23% among four stand ages (Supplementary Figure S6). The Alphproteobacteria contain the largest number of OTUs which was classified to seven families. The dominant bacterial species (i.e., the 30 most abundant OTUs) comprised Proteobacteria (31.9–46.5%), Acidobacteria (12.0–46.6%), and Actinobacteria (10.7–42.6%; Figures 4A,B, Supplementary Figure S7). There were no significant differences in bacterial phyla with a relative abundance >10% across all stand ages. Gemmatimonadetes, Chloroflexi, and Cyanobacteria, whose relative abundances were <10%, tended to decrease, whereas TM6 and OD1 showed an opposite trend along stand age. The relative abundance of TM7 was higher in mature plantations (planted 21–60 years ago) than in plantations of other stand ages.

We found significant difference in bacterial community among stand ages based on PERMDISP (Table 1). The soil bacterial communities under both young and near-mature plantations had significant difference with mature plantation, as well as over-mature plantation (Supplementary Table S2). Moreover, the PCoA plot showed that soil samples clustered tighter with increasing stand age (Figure 4C). Soil pH (*F* = 1.51, *p* = 0.016) and SOC content (*F* = 1.56, *p* = 0.012) were identified as drivers of the bacterial community structure based on RDA (Figure 4D).

**Table 1:** Significance test of the differences of centroids with the succession age.^a^

Centroid of <9 years communities	Centroid of 9–20 years communities	Centroid of 21–60 years communities	Centroid of >60 years communities	*F*	*p*
0.5008	0.4870	0.4262	0.4218	8.1067	0.001

^a^*Permutational analysis of multivariate dispersions (PERMDISP) was performed to test the significance of the difference*.

### Assembly Processes of Bacterial Communities Along Stand Age

**Figure 5:**
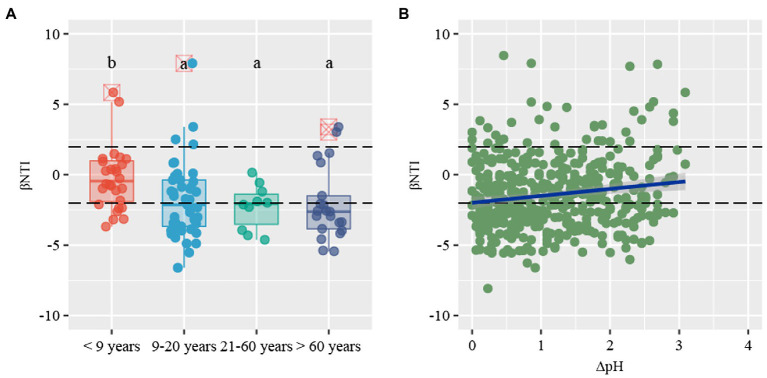
**(A)** Distribution patterns of β-nearest taxon index (βNTI) values along stand age. **(B)** The relationship between βNTI values and changes of soil pH. Horizontal dashed lines indicate lower and upper significance thresholds at −2 and +2, respectively.

β-nearest taxon index values provide insights into the potential roles of deterministic and stochastic forces in bacterial community dynamics. The βNTI distribution gradually shifted along stand age, from stochastic community assembly (|βNTI| < 2) to homogeneous selection (βNTI < −2; Figure 5A). Specifically, stochastic processes contributed to the community assembly in young plantations (53.6%), whereas deterministic process mostly contributed to assembly in near-mature (60.0%), mature (50.0%), and over-mature plantations (81.0%). The community assembly in young plantations was primarily governed by homogenizing dispersal (21.4%) and dispersal limitation (32.1%), respectively. Conversely, strong homogeneous selection of deterministic processes dominated the near-mature (51.1%), mature (50.0%), and over-mature plantations (71.4%; Table 2). The Mantel test results suggested that βNTI values were significantly affected by soil pH (*ρ* = 0.130, *p* < 0.05; Figure 5B, Supplementary Table S3).

**Table 2:** The relative contributions of ecological assembly processes across successional age.

Years	Variable selection	Homogeneous selection	Deterministic^a^	Homogenizing dispersal	Dispersal limitation	Stochastic^b^	Undominated
<9	0.107	0.250	0.357	0.214	0.321	0.536	0.107
9–20	0.089	0.511	0.600	0.200	0.111	0.311	0.089
21–60	0	0.500	0.500	0.300	0	0.300	0.200
>60	0.095	0.714	0.810	0.190	0	0.190	0

a *Deterministic = Variable selection + Homogeneous selection*.

b *Stochastic = Dispersal limitation + Homogenizing dispersal*.

### Global Co-occurrence Patterns of Bacterial Communities

**Figure 6:**
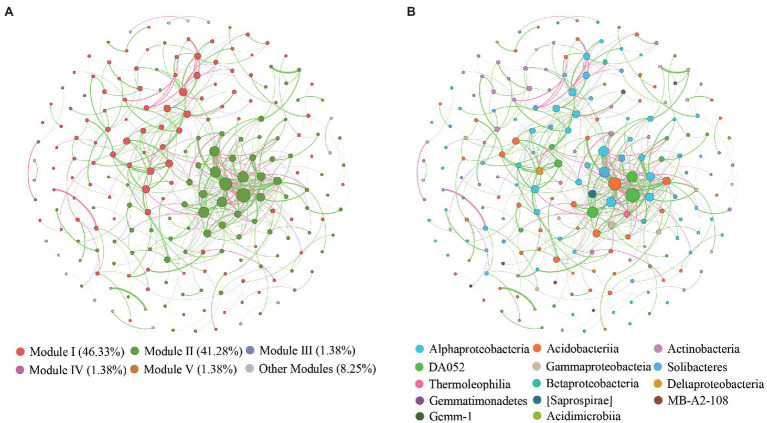
Co-occurrence patterns of OTUs in bacterial communities. Nodes are colored according to different **(A)** modularity classes and **(B)** class-level taxonomy.

**Figure 7:**
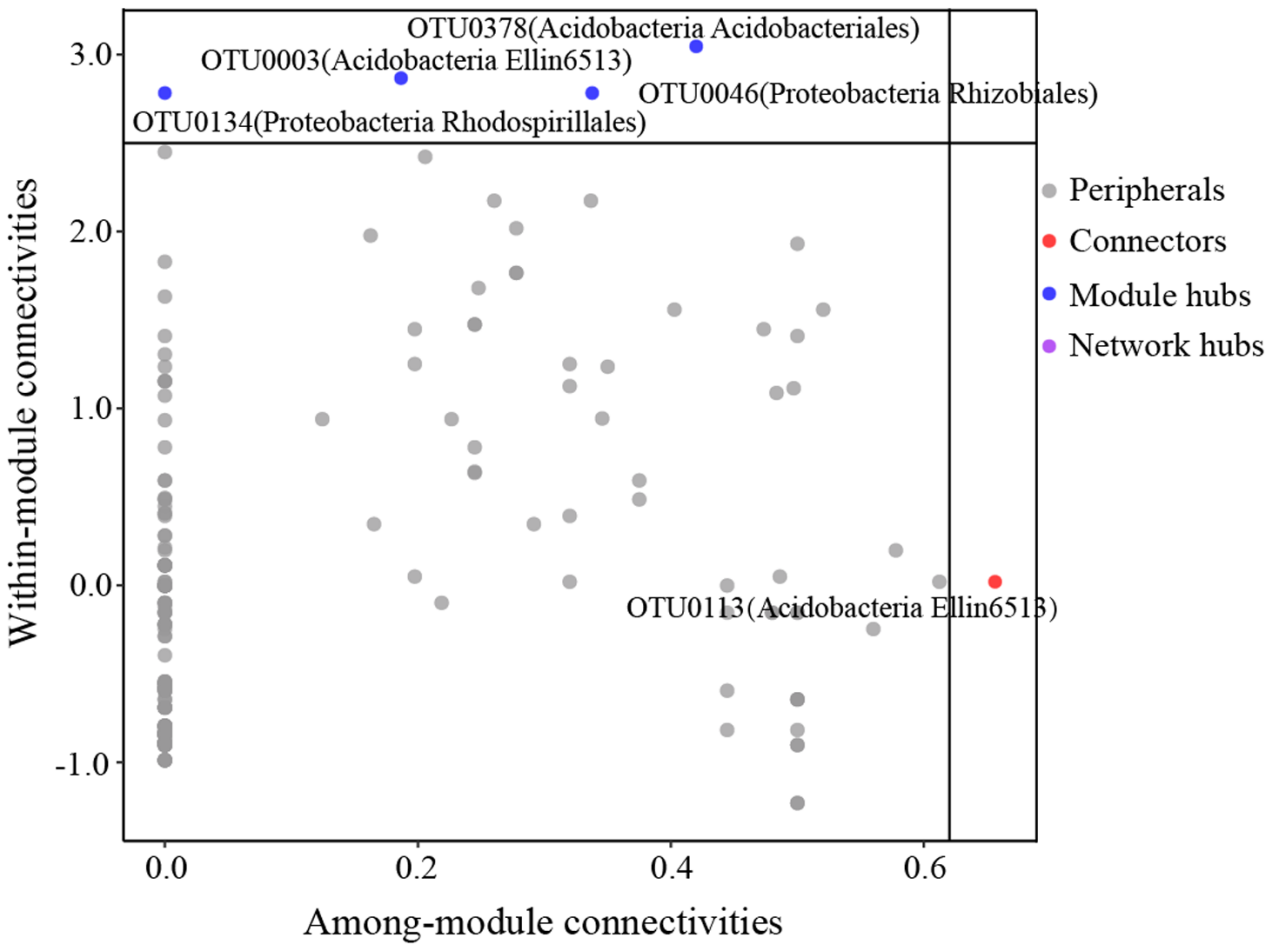
Z-P plot showing the classification of nodes to identify putative keystone species in the *C. oleifera* plantations. Each symbol represents an OTU. One connector was identified, assigned to Acidobacteria. There were four module hubs in the network, two belonging to Acidobacteria and two belonging to Proteobacteria.

The global co-occurrence network generated based on soil bacterial OTU profiles comprised 218 nodes and 478 links. The global network contained six modules, and two main modules accounted for 48.62 and 38.99% of the total number of nodes (Figure 6A). Alphaproteobacteria, Acidobacteria, Actinobacteria, and DA052 mainly occupied the nodes (Figure 6B). Sixty-eight percent of the links in the global co-occurrence network were positive. According to the Zi-Pi plot, most OTUs were identified as peripherals. Only OTU0113, assigned to Acidobacteria, served as a connector, and four OTUs, including OTU0046 (Proteobacteria), OTU0134 (Proteobacteria), OTU0003 (Acidobacteria), and OTU0378 (Acidobacteria), were module hubs (Figure 7). The topological properties of transitivity, average degree, and density tended to slightly decrease, whereas the average path length increased along stand age (Supplementary Table S4).

## Discussion

### Soil Microbial Resource Limitations Along Stand Age

Microbial C limitation is widespread in forest ecosystems (Soong et al., 2020). Although the SOC was gradually accumulated (Supplementary Table S5), we found that microbial C limitation progressively increased with increasing stand age. This is inconsistent with a previous finding that microbial C limitation decreased with increasing stand age in *Robinia pseudoacacia* planted forest (Zhong et al., 2020). One possible reason for this discrepancy is that microbial C limitation is, at least in part, determined by plant litter quality. Refractory organic materials from *C. oleifera* are more abundant in lignin than in cellulose (Hu et al., 2015), which result in a lower efficient C resource for soil microbe.

Our results indicated that microbial P limitation rather than microbial N limitation is common in the subtropical region, which was supported by the eco-enzymatic stoichiometry plot and vector analyses. Dissolved N reportedly is three times higher in subtropical areas than in temperate regions (Wu et al., 2019; Xiao et al., 2019), leading to the alleviation of microbial N limitation in those areas. Artificial N input in the initial stage of *C. oleifera* cultivation may also explain this result. Considering that P deficiency due to the strong adsorption of orthophosphates to soil aluminum (Al) and iron (Fe) oxides is common in subtropical regions, the widespread microbial P limitation observed in the soils evaluated in our study was not surprising. Notably, microbial P limitation tended to decrease along stand age, which is inconsistent with a previous finding that forest succession aggravated microbial P limitation (Huang et al., 2013). One possible explanation is that the continuous release of organic acids from *C. oleifera* roots can mobilize soil fixed P and alleviate microbial P limitation in more mature stands (Yuan et al., 2013).

### Soil Bacterial Community Changes Along Stand Age

Our results indicated that soil bacterial alpha diversity did not significantly change with stand age, which is inconsistent with a previous finding that the soil bacterial alpha diversity increased with succession age in a reforestation context (Jiao et al., 2018). This may be explained by plant diversity; as mentioned above, the resource diversity hypothesis states that diversified plant species provide various organic substrates and thus, diverse C resources, to the soil microorganisms (Liu et al., 2018). In contrast, in the *C. oleifera* plantations in our study, due to management practices such as weeding, only one species was present, providing only a simple carbon substrate for the soil microbes. Additionally, soil bacterial community was irrelevant to microbial C and P limitation when we analysis the effect of enzymatic stoichiometric parameters on soil bacterial community using the RDA selection, the possible reason could be that microbial metabolic limitation calculated by both soil bacterial and fungal enzyme activity.

Soil bacterial communities are strongly influenced by the plant communities in forest ecosystems (Dang et al., 2017). Our study indicated that the soil bacterial communities have significant change among stand ages, and the soil bacterial communities become more clustered in the later stage, suggesting that the special plant could shape soil bacterial community, because of the special plant has distinctive root characteristics including morphology, quantity, and exudates components (Wang et al., 2018). Previous studies revealed that root exudates in certain plant can modify soil bacterial community. For example, the root exudates of barrel clover and wheat enriched Proteobacteria and Actinobacteria in the rhizosphere (Haichar et al., 2008).

Besides Acidobacteria, Proteobacteria was reported to be the most abundant bacterial phyla in terrestrial soil (Wei et al., 2018), which is consistent with our study that Proteobacteria always was a major abundant phylum in the soil samples among stand ages. Interestingly, although the soil fundamental physic-chemistry parameters varied with stand age, such as SOC, the relative abundance of phylum Proteobacteria had no significant change along the stand age, suggesting that the Proteobacteria has strong sophisticated adaptations. Proteobacteria are also predominant in glacial ice and deep undersurface soils, suggesting that species within this phylum are highly resistant to various harsh environments (Shtarkman et al., 2013). Notably, Chloroflexi were more abundant in young stands than in older stands. Chloroflexi are reported to prefer nutrient-poor soils (Wang et al., 2019). Before the plantation of the *C. oleifera* stands evaluated in our study, the topsoil was widely destroyed by land leveling, which had caused runoff loss of soil nutrients and resulted in high microbial nutrient limitation in the young stands. High Gemmatimonadetes abundance was more often found in younger than in older stands. Gemmatimonadetes reportedly better prevail in dry soils than in moisture soils (Fawaz, 2013). The young *C. oleifera* plantations had lower vegetation coverage, and therefore, a lower ability to maintain soil moisture. Interestingly, we found that the relative abundances of some soil rare bacteria, such as TM7, TM6, and OD1, increased along stand age. Previous studies have shown that these species are widely detected in anaerobic environments (Winsley et al., 2014). Indeed, in the old *C. oleifera* plantations, the soil surface was covered with litter, which would have provided a more anaerobic soil condition.

### Assembly Processes and Co-occurrence Network of the Soil Bacterial Community

Stochastic and deterministic processes control the assembly of microbial communities along ecological succession (Fargione et al., 2003). We found that the soil bacterial community assembly of the *C. oleifera* plantations initially was governed by stochasticity. A possible reason may be that the strong soil disturbance before the establishment of the *C. oleifera* plantations destroyed the microbial community structure, resulting in a weak environmental filter. A previous study showed that stochastic processes dominated plant community assembly after fire disturbance (Måren et al., 2018). Therefore, plant-associated and soil microbes may undergo the same assemblage processes after intense disturbance. Interestingly, we found that the contribution of homogeneous selection was far more than the variable selection in all stand ages, which indicated that the bacterial community driven by consistent selective pressure of local environmental condition. A previous study demonstrated that deterministic processes are more likely to be driven by environmental gradients, for example, in soil pH and soil temperature (Jiao and Lu, 2020). In line herewith, we observed a decreasing trend in soil pH with stand age, and βNTI values were highly correlated with soil pH. Interestingly, a previous study showed that stochasticity drove soil bacterial community assemblage also under neutral pH (Tripathi et al., 2018). Therefore, the soil pH range may control soil bacterial community assembly. Considering that the fairly low soil pH in our studied ecosystem, a small decrease of pH in acid soil may provide a strong selection pressure on certain bacterial species. Furthermore, according to the established conceptual model, the stochastic processes governed the bacterial community initially. With the microbial succession, consistent microbial interaction progressively altered shelter environment through products of microbial metabolism and the change of nutrients, which provided a selective pressure from stochastic processes to deterministic processes.

An ecological soil bacterial network can reflect the complex interactions of soil bacteria (Chow et al., 2014). We found more positive than negative links in our global co-occurrence network, suggesting a high level of cooperation between the soil bacterial species in the *C. oleifera* plantations. The main modules, including modules I and II, mainly comprised Proteobacteria and Acidobacteria. Modules I and II represented two ecological types (Ishimoto et al., 2021), suggesting that those species belong to Proteobacterial and Acidobacterial phyla could be located in different functional niche. Intriguingly, rare species were found to occupy several modules with a few nodes, which implied that these species exist in distinct ecological niches. Keystone species in our study belonged to Koribacteraceae, Hyphomicrobiaceae, and Rhodospirillaceae. Previous studies have demonstrated that Koribacteraceae, which can decompose complex carbon polymers, are widely distributed in woodlands (Ward et al., 2009). Additionally, Koribacteraceae are involved in iron redox reactions in iron-rich environments, which may have benefited their survival in the *C. oleifera* plantations in this study (Su et al., 2020). The *C. oleifera* plantations were located in subtropical regions, where the soil is rich in iron and low in C due to strong weathering and nutrient leaching. Hyphomicrobiaceae and Rhodospirillales reportedly commonly occur in hypoxic environments, and species of these classes can photosynthesize and are involved in oxidative metabolism (Anderson et al., 2011). The possible reasons for the occurrence of these species may be that the compact soil structure led to a low soil oxygen content, and the occasional heavy rains diminished soil aeration.

Stand age contributes to the topological features of bacterial co-occurrence networks in *Cunninghamia lanceolata* planted forests (Cao et al., 2020). We observed a loss of network complexity with stand age, suggesting a progressively weaker interaction between bacteria. The soil disturbance before the establishment of the *C. oleifera* plantations may have contributed to a more diverse array of ecologically functional groups, which would imply more potential interactions. Subsequent the progressive acidification of soil in the *C. oleifera* ecosystem would have decreased the microbial growth condition along the stand age, which may have inhibited the activity of soil bacteria, resulting in weaker cooperation among the bacterial species.

## Conclusion

This study investigated soil microbial limitation and soil microbial community dynamics along stand age in a planted *C. oleifera* forest in a subtropical region in China. Our study provided solid evidence that P and co-C and P limitations were dominant soil microbial resource limitations in this ecosystem. Soil microbial P limitation tended to decrease with stand age. Microbial community assembly tended to shift from stochastic to deterministic processes along stand age, and soil pH was identified as filtering factor for soil bacterial community assembly. Additionally, we found that soil bacteria likely experienced more extensive nutrient depletion in young stands, suggesting that low soil fertility promotes microbial cooperation to obtain essential nutrients. Our findings shed light on microbial limitations and assemblage patterns in planted forest ecosystems and improve our knowledge regarding the drivers of community assembly along stand age. In addition, our finding suggested the importance of proper nutriment management, especially for P, in *C. oleifera* plantations in subtropical area.

## Data Availability Statement

The data sets presented in this study can be found in online repositories. The names of the repository/repositories and accession number(s) can be found in the article/Supplementary Material.

## Author Contributions

YH and YS designed the study. YH and CD collected the soil samples. HQ, CD, QS, and SD performed physicochemical, enzymatic, and bacterial analysis of all soil samples. HQ and LC analyzed the data with help from YR, YH, XC, JW, LM, HQ, and LC wrote the paper with inputs from all co-authors.

## Funding

This research was supported by grants from the National Key Research Program (2017YFC0505503); Science and Technology Innovation Program of Hunan (2020NK2005); National Science Foundation (41601260); and Natural Science Foundation of Guangxi (2018GXNSFAA138020).

## Conflict of Interest

The authors declare that the research was conducted in the absence of any commercial or financial relationships that could be construed as a potential conflict of interest.

The reviewer WC declared a shared affiliation with one of the authors, LM, to the handling editor at time of review.

## Publisher’s Note

All claims expressed in this article are solely those of the authors and do not necessarily represent those of their affiliated organizations, or those of the publisher, the editors and the reviewers. Any product that may be evaluated in this article, or claim that may be made by its manufacturer, is not guaranteed or endorsed by the publisher.

## Footnotes

^1^ https://itol.embl.de/

^2^ https://cran.r-project.org/
