# Supplementary material for: Soil Microbial Resource Limitations and Community Assembly Along a Camellia oleifera Plantation Chronosequence
Source: Front Microbiol. 2021 Dec 2;12:736165. doi: 10.3389/fmicb.2021.736165 (PMC8675945; doi:10.3389/fmicb.2021.736165)

Soil microbial resource limitations and community assembly along an age gradient of a subtropical planted *Camellia oleifera* forest

Hang Qiao, Longsheng Chen, Yajun Hu, Chenghua Deng, Qi Sun, Shaohong Deng, Xiangbi Chen, Li Mei, Jinshui Wu and Yirong Su

Supplementary information

Table S1. Enzymes included in this study.

Enzyme	Abbreviations	EC[a]	Function
β-D-cellobiosidase	CBH	3.2.1.91	Cellulose degradation: hydrolyses cellobiose dimers from non-reducing ends of cellulose molecules
β-1, 4-glucosidase	BG	3.2.1.21	Cellulose degradation: hydrolyses glucose from cellobiose
L-leucine aminopeptidase	LAP	3.4.11.1	Proteolysis: hydrolyses leucine and other hydrophobic amino acids from the N terminus of polypeptides
β-N-acetylglucosaminidase	NAG	3.2.1.14	Chitin and peptidoglycan degradation: hydrolyses glucosamine from chitobiose
Acid phosphatase	AP	3.1.3.2	Hydrolyses phosphate from phosphosaccharides and phospholipids

Note: [a] Enzyme commission classification (Sinsabaugh et al., 2008, 2009).

Table S2. Significant differences of soil bacterial community between stand age.

	P
< 9 years *vs* 9-20 years	0.519
< 9 years *vs* 21-60 years	0.005
< 9 years *vs* > 60 years	0.001
9-20 years *vs* 21-60 years	0.046
9-20 years *vs* > 60 years	0.008
21-60 years *vs* > 60 years	0.852

Table S3. Mantel tests with Spearman's rank correlations between environmental factors (Euclidean distance) and βNTI of bacterial communities with 999 permutations. SOC: soil organic carbon, TN: total nitrogen, TP: total phosphorous, Olsen-P: Olsen phosphorous. ($P < 0.05$).

Variables	ρ (Mantel test)	*P*
pH	0.130	0.017
SOC	-0.021	0.573
TN	-0.080	0.785
TP	0.142	0.117
Olsen-P	0.084	0.247

Table S4. Topological properties of networks at different stand ages in *C. oleifera* plantation. Different letters denote significant difference among stand ages ($P < 0.05$).

Stand age	Edges	Nodes	Transitivity (Trans)	Average Degree (AD)	Betweenness centrality (BC)	Average path Length (GD)	Density (DS)
< 9 years	245.13±63.77a	131.00±28.37a	0.37±0.04b	3.72±0.25b	0.19±0.06a	4.37±0.33a	0.03±0.01b
9 – 20 years	263.20±42.34a	143.70±19.80ab	0.35±0.05ab	3.67±0.31b	0.18±0.05a	4.54±0.28ab	0.03±0.01ab
21 – 60 years	269.80±45.71a	156.80±18.62b	0.35±0.03ab	3.43±0.28ab	0.17±0.05a	4.79±0.17b	0.02±0.00a
> 60 years	251.29±28.78a	152.57±9.64ab	0.32±0.02a	3.29±0.24a	0.19±0.049a	4.82±0.36b	0.02±0.00a

Different lowercase letters in the same column indicated the significance between different stand age ($P < 0.05$).

Table S5. Variations of soil physicochemical properties at different stand ages in *C. oleifera* plantation. Different letters denote significant difference among stand ages.

Stand age	pH	SOC(g/Kg)	TN(g/Kg)	TP(g/Kg)	Olsen-P(mg/Kg)
< 9 years	4.39±0.20a	10.52±3.97a	1.14±0.29a	0.35±0.21a	6.54±5.78a
9 – 20 years	4.40±0.15a	17.68±5.83b	1.50±0.37ab	0.40±0.19a	5.70±3.42a
21 – 60 years	4.32±0.20a	20.71±2.67b	1.75±0.28b	0.30±0.10a	4.21±0.95a
> 60 years	4.24±0.16a	23.11±5.77b	1.85±0.50b	0.34±0.14a	5.10±1.39a

SOC, soil organic carbon; TN, total nitrogen; TP, total phosphorus; Olsen-P, Olsen phosphorous. Different lowercase letters in the same column indicated the significance between different stand age ($P < 0.05$).

Fig. S1. *P* value for the KS, SSE, and NNSD tests of the exponential distribution generated by random matrix theory.

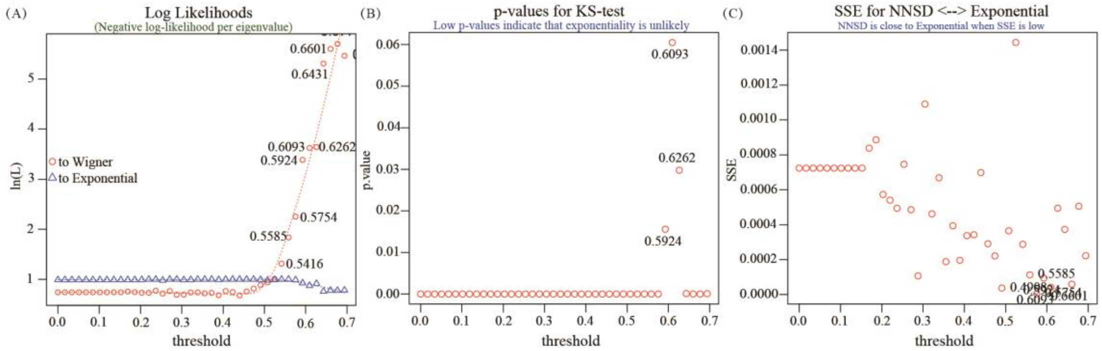

Figure S2. Variations of soil enzyme activities at different stand ages in C. oleifera plantation. Note: (A) CBH; (B) BG; (C) LAP; (D) NAG; (E) AP. Different letters denote significant difference among stand ages ($P < 0.05$).

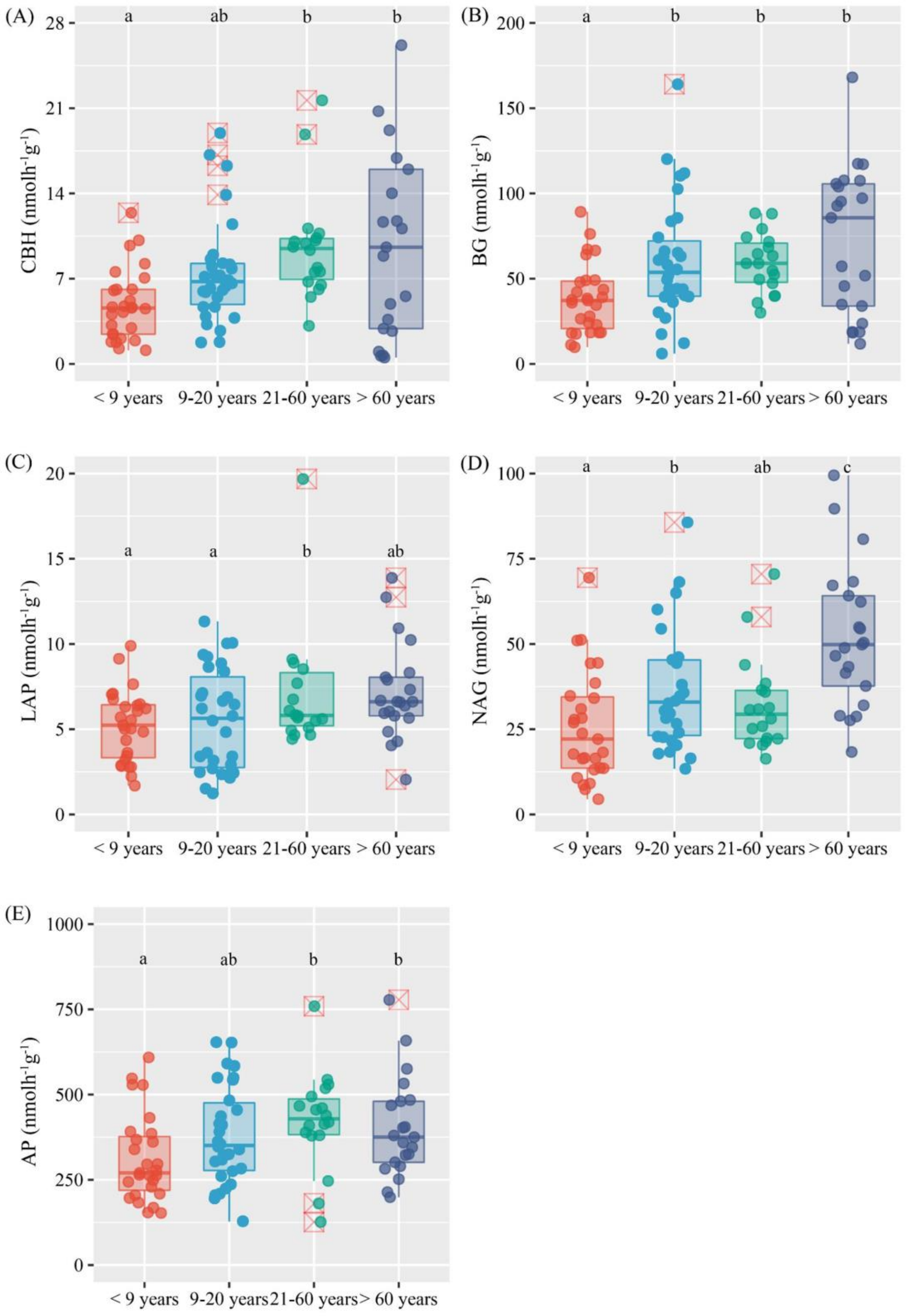

Fig. S3. Variations of soil enzymatic stoichiometry at different stand ages in *C. oleifera* plantation Note: (A), (CBH+BG)/(NAG+LAP); (B), (CBH+ BG)/AP; (C), (NAG + LAP)/AP. Different letters denote significant difference among stand ages ($P < 0.05$).

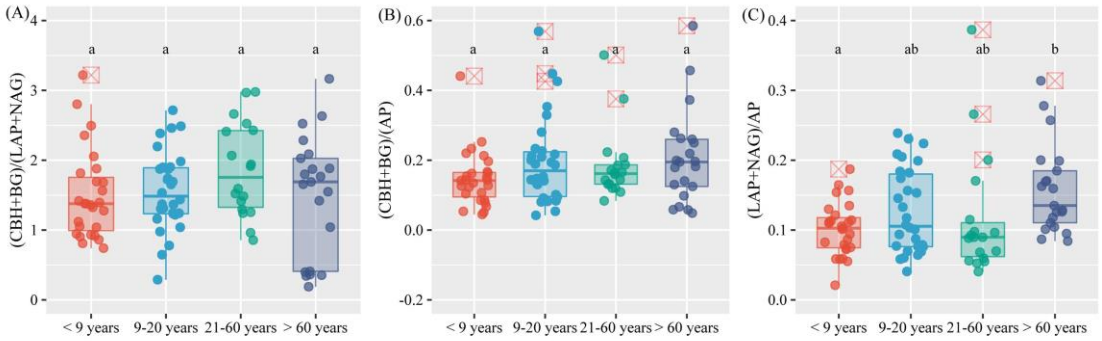

Fig. S4. Relationships between (A-C) vector A and soil organic carbon, total nitrogen or total phosphorus, and between (D-F) vector L and soil organic carbon, total nitrogen or total phosphorus. All P values of the regression analysis were < 0.05. SOC: soil organic carbon, TN: total nitrogen, TP: total phosphorous.

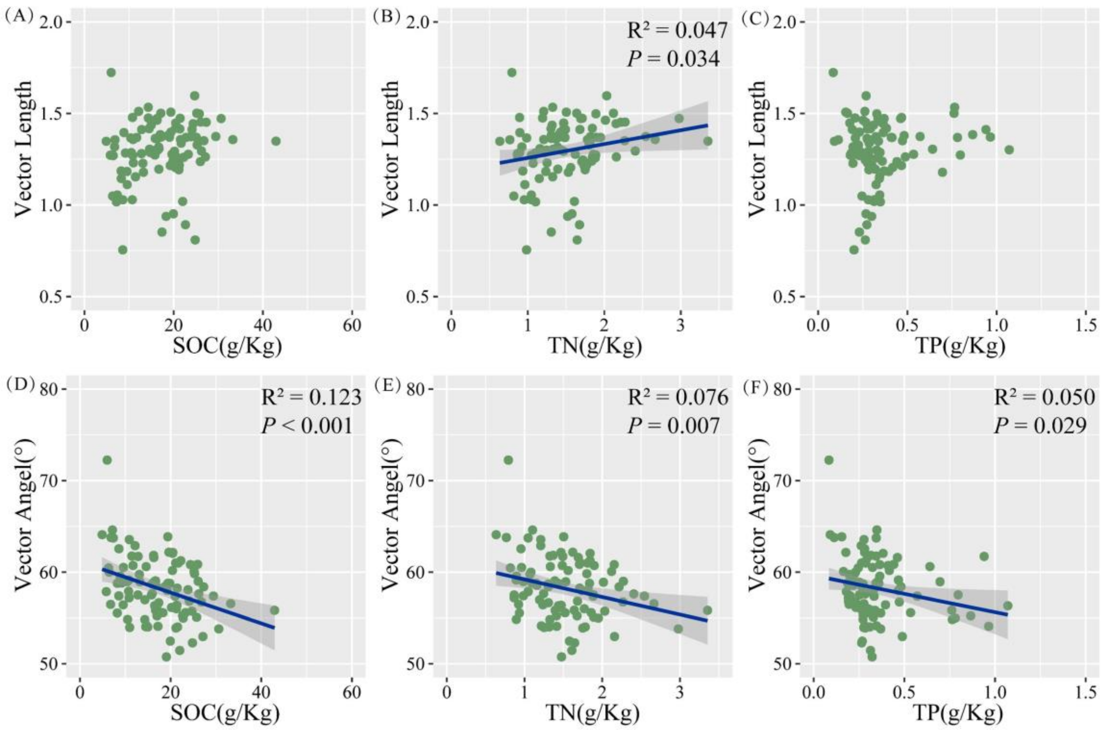

Fig. S5. Variations of soil bacterial alpha diversity at different stand ages in *C. oleifera* plantation Note: (A), richness; (B), Shannon diversity.

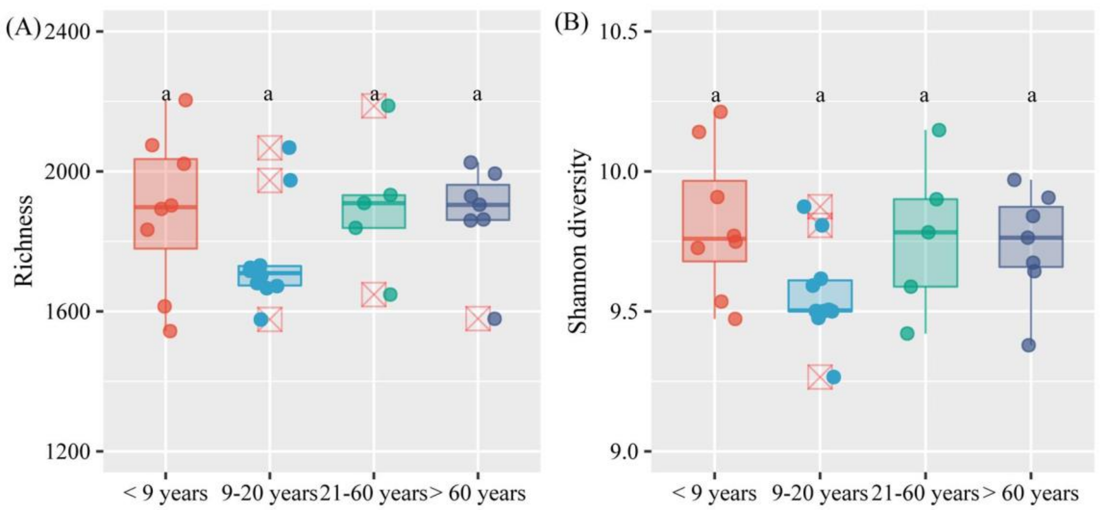

Fig. S6. Phylogenetic tree, taxonomic composition, and distribution patterns of C. oleifera dominant taxa.

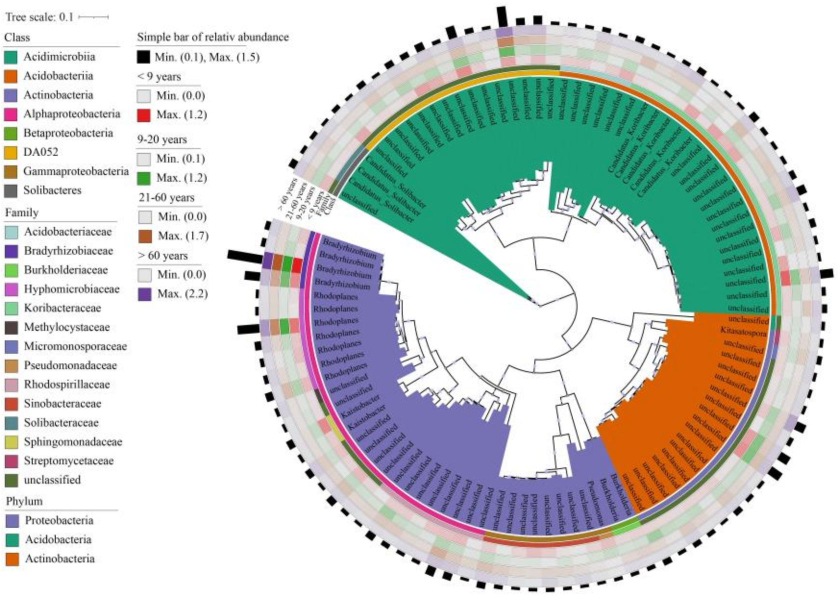

Fig. S7. Boxplot of the abundance of the 30 most abundant OTUs.

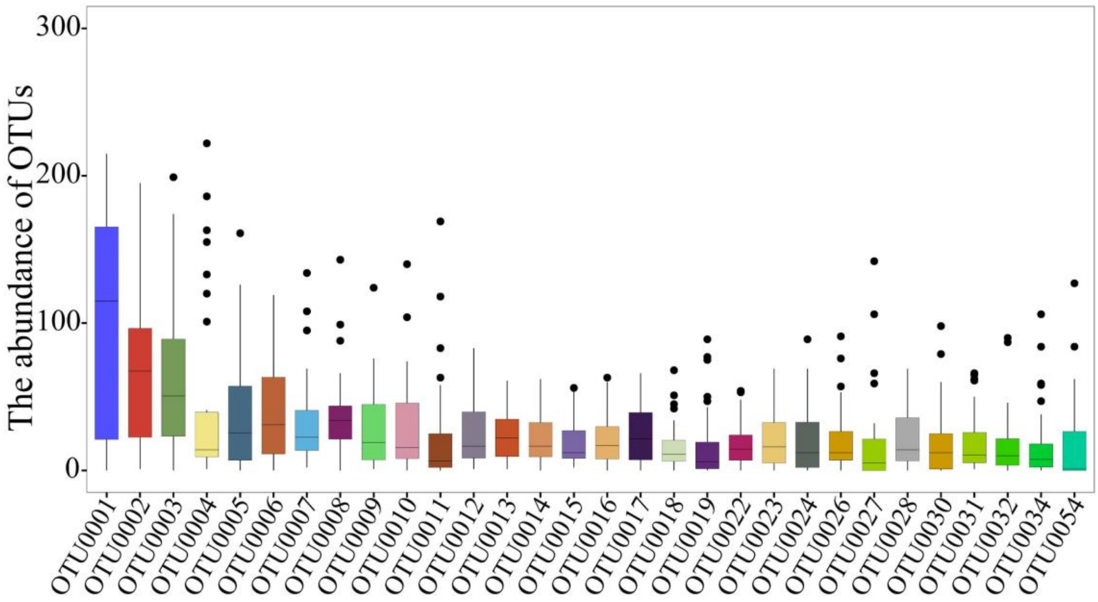

Supplement: Supplementary file 1 [file Presentation_1.pdf]
